# Practical Chemistry for Students and Physicians—Chapter Fifth

**Published:** 1883-11

**Authors:** 


### Editorial.

### PRACTICAL CHEMISTRY FOR STUDENTS AND PHYSICIANS—CHAPTER FIFTH.

(Continued from October Register, Vol. III, Page 27.)

Having thus briefly disposed of all that is important to be said in this place of the four elementary gases, we will present the subject of *chemical combination.*

This is one of the new features of the modern chemistry, which has so greatly enhanced, not only the facilities for its successful study and investigation, but given it a higher claim to be placed permanently in the domain of science.

At the dawn of the present century, it had been ascertained through the labors of several distinguished investigators, that the following law must be accepted as a truth in chemistry: "When *compounds* unite to form definite chemical substances, they always combine in the same proportions."

Soon after it was announced, for the first time, by John Dalton, a distinguished English philosopher, that *elements* unite, always in the same proportions to form definite substances. This law is obviously fundamental, and led, at once, to the statement of the higher generalization, embracing the phenomena of both laws, viz: A definite chemical compound always contains the same elements and in the same proportions.

For convenience, this generalization may be stated in two separate laws, to be given presently. Continuing his investigations, Dalton shortly announced another law of chemical combination no less new and wonderful, viz: When one element unites with another in two or more proportions, the higher proportions are always a simple multiple of the first or lower proportion.

From these two, or rather three generalizations, it was easy to deduce a fourth and a fifth, viz: First, when two elements unite in certain proportions, respectively, with a third, these are the same proportions in which they will unite with each other; second, the weight of any definite chemical compound is equal to the sum of the weight of its constituents.

We will place these laws in their logical order. The first may be named the law of *constant composition. The same chemical compound is always composed of the same constituents.*

The second may be named the law of *constant proportion. When elements* or substances unite *to form a definite compound, they always combine in the same proportions.*

The third law is called the law of *multiple proportions. When one element unites with another in two or more proportions, the higher proportions are always a simple multiple of the first or lower proportions.*

The fourth law is the law of *reciprocal proportion. When two elements unite in certain proportions, respectively, with a third, these are the same proportions in which they will combine with each other.*

The fifth is the law of *componnd proportion. The weight of any definite chemical compound is equal to the sum of the weight of its constituents.*

In order to establish his two fundamental laws, Dalton appropriated and turned to account, for the first time in its history, an old theory of the Greek philosophy, which taught that matter is not infinitely divisible, but composed of indefensibly minute and indivisible particles or atoms.

He proceeded farther, and pictured these atoms as having not only an appreciable existence, but each its own special weight and unchangeable characteristics—that where different kinds of matter unite to form compounds, it was not by a vague interpenetration of the combining atoms, but by their gathering or aggregation into indivisible bundles called molecules—little masses.

It is thus seen that chemical action is directed by a force as exact, definite and irresistible in its sphere as that which sustains the planets in their orbits and holds them in their proper places. Were any other ingredients than hydrogren and oxygen combined to form water, no water would ever be formed, and were those gases used for the same purpose in less proportion than two of hydrogen and sixteen of oxygen by weight, there would be an equal failure to form water. Nothing is ever lost or wasted in the economy of nature, when left to apply her own laws.

Under the law of multiple proportion the best illustration is found in the five remarkable oxides of nitrogen. They are thus written: $N_2O$; $N_2O_2$; $N_2O$ ; $N_2O_4$, and $N_2O_5$. It is plain that the first formula contains the lowest proportion of oxygen in its combination with nitrogen, and that the other compounds have it in the proportions of 2, 3, 4 and 5, each number being a simple multiple of the quantity of oxygen in the first compound.

The illustrations of the law of reciprocal proportion are numerous in chemistry. Take, for example, oxygen combining with hydrogen in the proportion of 16, by weight, to form water. But nitrogen combines also with hydrogen in the proportion of 14, to form ammonia gas; hence 16 and 14 are just the reciprocal proportion in which oxygen and nitrogen will unite with each other to form an oxide.

The molecular weight of any compound in chemistry illustrates the law of compound proportion. Take the weight of a molecule of common salt, NaCl; the atomic weight of the sodium, Na, is 23, and that of the chlorine, Cl, is 35.5; hence the chemical weight of a molecule of sodium chloride is $23 \bowtie 35.5 = 58.5$.

